# Advances in Machine Learning-Aided Thermal Imaging for Early Detection of Diabetic Foot Ulcers: A Review

**DOI:** 10.3390/bios14120614

**Published:** 2024-12-13

**Authors:** Longyan Wu, Ran Huang, Xiaoyan He, Lisheng Tang, Xin Ma

**Affiliations:** 1 Academy for Engineering and Technology, Yiwu Research Institute, Fudan University, Shanghai 200433, China; 22210860027@m.fudan.edu.cn (L.W.); xiaoyanhe@rizt.ac.cn (X.H.); tangls@rizt.ac.cn (L.T.); 2 Center for Innovation and Entrepreneurship, Taizhou Institute of Zhejiang University, Taizhou 318000, China; 3 Shanghai Sixth People’s Hospital, Shanghai Jiao Tong University, Shanghai 200233, China

**Keywords:** diabetic foot ulcer, thermal imaging, two-dimensional signal processing, machine learning

## Abstract

The prevention and early warning of foot ulcers are crucial in diabetic care; however, early microvascular lesions are difficult to detect and often diagnosed at later stages, posing serious health risks. Infrared thermal imaging, as a rapid and non-contact clinical examination technology, can sensitively detect hidden neuropathy and vascular lesions for early intervention. This review provides an informative summary of the background, mechanisms, thermal image datasets, and processing techniques used in thermal imaging for warning of diabetic foot ulcers. It specifically focuses on two-dimensional signal processing methods and the evaluation of computer-aided diagnostic methods commonly used for diabetic foot ulcers.

## 1. Introduction

Diabetes mellitus (DM) is one of the most formidable challenges confronting public health in the 21st century, affecting millions worldwide. DM, a metabolic disorder marked by chronic hyperglycemia, stems from either a deficiency in insulin production or the body’s inability to use insulin effectively. This imbalance disrupts normal glucose metabolism, leading to a wide range of complications if not adequately managed. According to the International Diabetes Federation (IDF), approximately 9.3% of the global population was affected by DM in 2019. Alarmingly, this prevalence is projected to rise to 10.2% by 2030 and 10.9% by 2045, driven by lifestyle changes, population aging, and other contributing factors [1].

Among the complications associated with DM, diabetic foot ulcer (DFU) stands out as one of the most common, severe, and debilitating. DFUs develop in approximately 25% of diabetic patients over the course of their illness [2,3]. These ulcers, which typically form due to a combination of neuropathy, ischemia, and infection, result in open sores or wounds on the foot that are slow to heal. If left untreated, DFUs can progress to severe infection or gangrene, necessitating limb amputation in extreme cases. Indeed, many patients with ulcerative foot lesions face amputation within four years of DFU onset [4,5].

This high rate of amputation not only imposes a heavy psychological and physical toll on patients but also translates into substantial healthcare costs, often exacerbated by high recurrence rates and the necessity of prolonged treatments. The economic burden associated with DFU is considerable, encompassing costs for hospitalization, surgery, wound care, and rehabilitation. Furthermore, DFUs contribute significantly to mortality rates in diabetic populations, with severe cases leading to death in extreme instances [6].

The increasing prevalence of DFUs over recent decades presents a growing challenge for healthcare systems worldwide. With the aging population and the rapid rise in DM cases, healthcare providers are confronted with the dual challenge of providing both preventive care and advanced treatments for DFU. In light of these challenges, early detection and timely intervention in foot ulcer development are critical. Preventive strategies, especially those enabled by advancements in medical imaging and machine learning, are essential for reducing the incidence and severity of DFUs. By focusing on early identification and effective management, healthcare systems can mitigate the severe outcomes of DFUs, including amputation and death, thereby enhancing the quality of life for diabetic patients and alleviating the long-term burden on global healthcare resources [7]. 

The metabolic disturbances characteristic of diabetes result from a partial or complete deficiency in insulin, a crucial polypeptide hormone responsible for regulating blood glucose levels. Insulin deficiency, coupled with the body’s impaired response to it, causes persistent hyperglycemia, which initiates a cascade of adverse effects at the cellular and molecular levels. High blood glucose levels lead to non-enzymatic glycosylation, a process in which excess glucose binds to proteins, lipids, and nucleic acids, forming advanced glycation end-products (AGEs). These AGEs disrupt the structural integrity and function of vascular tissues, leading to endothelial dysfunction and impairing normal blood flow [8].

The vascular abnormalities that ensue from diabetes manifest as both microvascular and macrovascular complications, impacting nearly every major organ in the body. Microvascular complications primarily affect the small blood vessels and are associated with conditions like diabetic retinopathy, nephropathy, and neuropathy. Retinopathy can lead to blindness, while nephropathy, affecting the kidney’s filtration ability, often progresses to chronic kidney disease and ultimately end-stage renal failure if untreated. Macrovascular complications, on the other hand, involve larger blood vessels, resulting in an increased risk of coronary artery disease, cerebrovascular disease, and peripheral artery disease. These cardiovascular conditions are leading causes of mortality and morbidity in diabetic populations [9].

Among these complications, diabetic neuropathy poses a particularly severe risk, especially for the lower limbs. Neuropathy, resulting from prolonged high blood glucose, leads to structural damage and functional impairment of the peripheral nerves, particularly in the feet and lower extremities. This nerve damage reduces sensory perception, often causing pain, tingling, or numbness in the affected areas. As neuropathy progresses, patients lose the ability to sense temperature changes, pressure, and minor injuries, making them vulnerable to unnoticed injuries or infections. Without prompt treatment, these minor injuries can escalate, causing deep tissue damage and bacterial infections that may require surgical intervention [10].

In addition to neuropathy, ischemic changes in the vascular system contribute to diabetic foot ulcers (DFUs). The restricted blood flow to the extremities, combined with impaired nerve function, makes wound healing slower and more challenging. This creates an environment where bacterial infections can thrive, leading to cellulitis, abscess formation, and even gangrene in severe cases. Consequently, most DFUs are the result of a combination of neuropathic and ischemic changes, which not only contribute to pain and numbness but also pave the way for potentially life-threatening infections and extensive tissue destruction [11].

**Figure 1:**
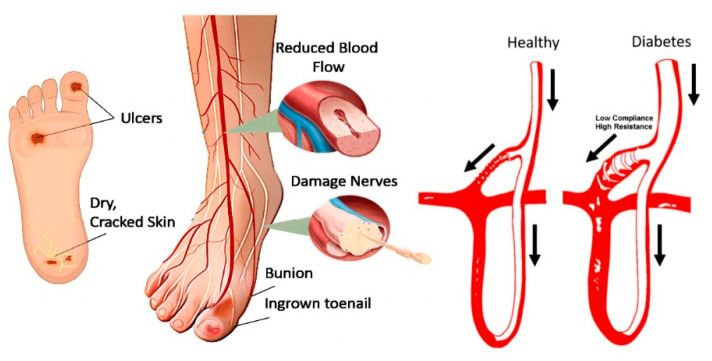
Pathogenesis of diabetes foot ulcer [12]. The arrows indicate the direction of blood flow, comparing a healthy foot to a diabetic foot, which is prone to ulceration due to disruptions in the microcirculatory system.

Given these factors, diabetic foot ulcers exemplify the broader pathogenesis of diabetes-related complications. The interplay between vascular impairment, nerve damage, and impaired healing underlines the critical need for early intervention and effective management strategies. Figure 1 illustrates various complications and the pathophysiology of diabetic foot ulcers, highlighting the complex interaction between neuropathy, ischemia, and infection in diabetes. This complex pathogenesis underscores the importance of preventive care and advanced diagnostic techniques, such as thermal imaging, to detect early signs of DFUs before they progress to severe stages.

Early identification of ischemic or neuropathic lesions in diabetic feet is critical to implementing timely prevention and diagnosis strategies for diabetic foot ulcers (DFUs), significantly lowering the risk of progression to foot amputation. Diabetic neuropathy and peripheral artery disease (PAD) often disrupt blood flow and nerve function, creating an imbalance in foot temperature that can signal underlying complications. This temperature imbalance is a key clinical indicator of diabetic neuropathy and is typically observed as localized areas of increased warmth or coolness in response to neuropathic damage, inflammatory processes, or compromised circulation.

In healthy individuals, temperature variation between regions of the feet is usually minimal, with differences generally remaining below 1 °C. However, in individuals with diabetic neuropathy or other complications, temperature differences exceeding this threshold can indicate pathological changes. Research has shown that a temperature increases of approximately 2.2 °C in specific foot regions is often a precursor to DFU development. Such temperature elevations may result from heightened metabolic activity due to inflammation, impaired vasodilation, or the body’s response to infection, all of which indicate the onset of a pathological condition.

Recently, infrared thermography has grown in prominence for use in biological applications including skin cancer diagnosis [13], pressure ulcers [14], breast cancer [15], and so on. Thermal imaging provides a non-invasive, efficient means of detecting these subtle temperature fluctuations and identifying high-risk areas before ulcers form. By mapping and analyzing these thermal patterns, clinicians can detect early signs of neuropathy or ischemia, facilitating timely intervention and thereby significantly reducing DFU incidence. Continuous monitoring and assessment of thermal patterns allow for more personalized care, enabling healthcare providers to identify when intervention is necessary to prevent the development or worsening of ulcers. Furthermore, integrating thermal imaging into regular diabetic foot assessments can help reduce hospitalization rates, minimize the need for aggressive treatments, and improve overall patient outcomes by catching potential DFUs early in their course.

In recent years, computer-aided diagnostic technology has been extensively explored for its potential to detect diabetic foot ulcers (DFUs) using infrared thermal imaging (IRT) to measure plantar temperature [16]. IRT offers a powerful, non-invasive means of assessing temperature distribution across the plantar surface, which is highly relevant for detecting early signs of diabetic neuropathy and vascular dysfunction—key contributors to DFU [17,18]. Traditional methods of DFU diagnosis rely heavily on clinicians’ subjective interpretation, which can vary based on experience and may miss subtle temperature variations that could indicate impending complications. Computer-aided diagnostic tools overcome this limitation by providing objective, consistent, and precise measurements, improving the sensitivity and specificity of DFU diagnosis.

The fundamental principle behind infrared thermal imaging lies in capturing the infrared radiation naturally emitted by the human body. Using advanced infrared lenses, IRT systems accurately measure the surface temperature of the foot and generate a thermogram, a visual representation that displays temperature variations across different regions of the foot. This thermogram enables the detection of temperature changes, even those arising from early functional metabolic abnormalities, before any visible structural abnormalities or physical symptoms appear. By revealing abnormal thermal patterns linked to inflammation, neuropathy, or reduced blood flow, IRT offers a valuable diagnostic window for anticipating potential DFU development well before ulceration becomes clinically apparent [19].

Thermal imaging technology boasts remarkable sensitivity, with a temperature measurement accuracy of 0.03–0.1 °C, allowing it to capture minute temperature fluctuations across thousands of pixels in the inspected area. This high precision supports detailed thermal analysis over a short time span, delivering rapid, accurate results that are critical for early detection and intervention. Furthermore, IRT provides a non-contact, non-invasive approach, reducing the discomfort and risk of contamination for patients, especially those with open wounds or fragile skin. Its wide temperature measurement range, coupled with its ability to produce intuitive, vivid thermograms, enables healthcare providers to visualize and track changes over time easily [20].

**Figure 2:**
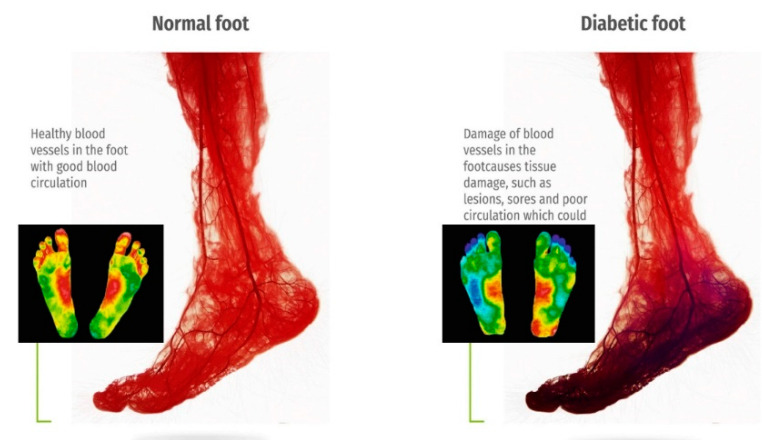
The contrast diagram of blood circulation and thermography of normal and diabetes feet [20,21].

The versatility and ease of use of thermal imaging make it well-suited for both clinical settings and remote monitoring applications. As the prevalence of diabetes rises globally, the demand for accessible, reliable, and cost-effective diagnostic solutions grows correspondingly. IRT, integrated with machine learning algorithms, can further enhance diagnostic accuracy by identifying complex patterns and trends in temperature distribution, paving the way for personalized preventive care. Figure 2 presents a comparative thermographic image of blood circulation in healthy versus diabetic feet, underscoring the visible differences that thermal imaging can detect. By capturing these essential diagnostic insights, thermal imaging can support the early diagnosis of DFUs, helping to reduce amputation rates and improve outcomes for diabetic patients [21].

## 2. Thermal Imaging Data Sets for Diabetic Foot Ulcers

### 2.1. INAOE Thermal Imaging Diabetic Foot Ulcer Dataset

In recent years, the number of diabetic foot cases has significantly increased, posing an increasing burden on healthcare systems and sparking interest in DFU research. Currently, one of the largest DFU detection-oriented datasets is from the Diabetic Foot Ulcer Challenge (DFUC2020). The DFUC2020 dataset consists of 4000 RGB images, with 2,000 images used for the training set and the other 2000 for the test set [22].

**Figure 3:**
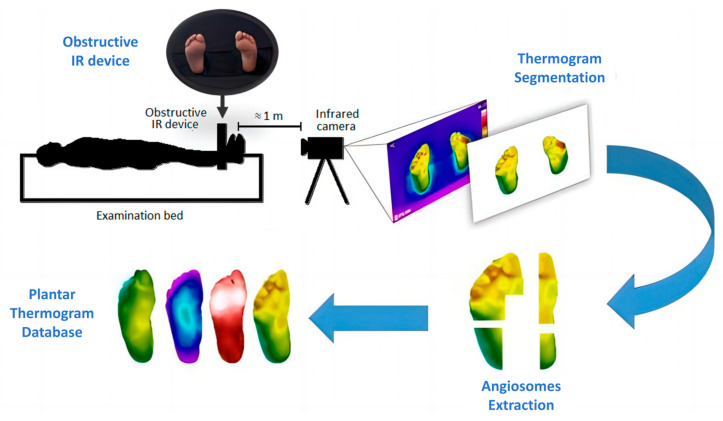
INAOE: Thermal Imaging Diabetic Foot Ulcer Dataset [23]. Perform thermogram segmentation and angiosomes feature extraction on the collected images to obtain the database.

There are relatively few datasets of plantar thermal images, with the mainstream dataset being the Thermal Imaging Diabetic Foot Ulcer (INAOE) Dataset released in December 2019 [23] (Figure 3). It contains thermograms of 122 diabetic patients and 45 non-diabetic subjects. This dataset can be used to study the temperature distribution of plantar regions and is widely used for classification tasks.

### 2.2. Methods to Improve Model Generalization in Data-Limited Scenarios

#### 2.2.1. Data Augmentation

Publicly accessible thermal imaging datasets for DFU are limited due to the high cost of acquiring medical images, the low frequency of certain pathologies, and the scarcity of labeled images. Considering the high generalization accuracy of effective analytical methods like deep learning networks [24], large amounts of data are required to train hundreds or thousands of internal parameters [23,25]. To counter this challenge, data augmentation techniques [26] and various preprocessing methods [27,28], such as data annotation, alignment, and feature extraction [29,30], have been applied to enhance and optimize datasets.

**Figure 4:**
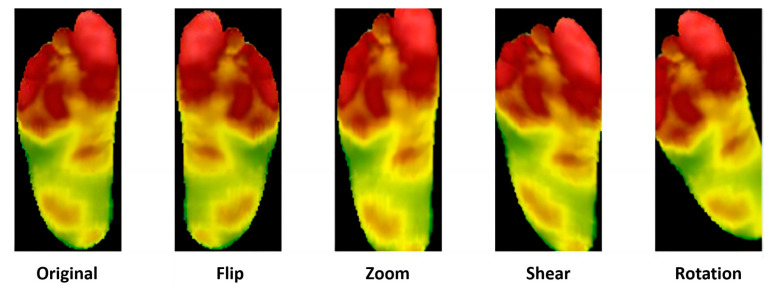
A demonstration of data augmentation [32].

Data augmentation can expand datasets without significantly increasing the risk of overfitting. For each image in the original set, data augmentation generates multiple slightly different versions. Techniques include rotating, scaling, flipping, and cropping images to create new ones, as shown in Figure 4 [31].

**Figure 5:**
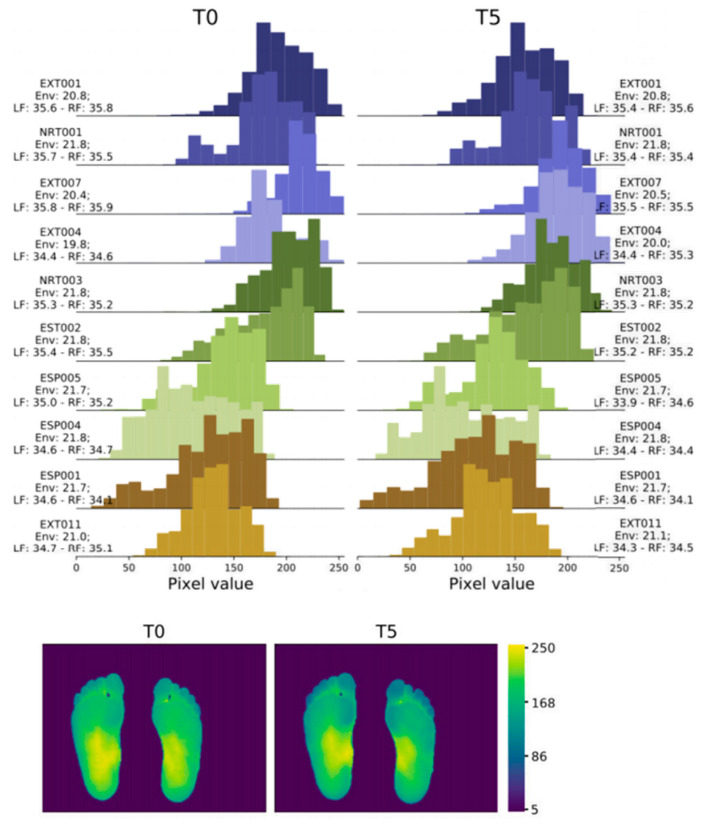
Histograms and heatmaps of a pair of images (T0 and T5) of the same subject extracted from the IACTEC dataset [33].

In 2022, Abian Hernandez-Guedes [33] proposed a technique that combines datasets to achieve data augmentation by applying preprocessing and histogram matching to increase image contrast. This approach established the IACTEC dataset, achieving over 95% accuracy, and using ResNetV2, even 100% accuracy (Figure 5). This data set uses 74 infrared thermal images from the technical center of the Instituto de Astrofísica de Canarias (IAC), collected from 37 non diabetes volunteers, including 15 women and 22 men, with an average age of 40 ± 8 years and between 24 and 60 years old. The images were obtained using a TE-Q1 Plus thermal imager from Thermal Expert (i3system Inc., Daejeon, Republic of Korea) and saved in a 16-bit PNG format with a spatial resolution of 384 × 288 pixels. Two sets of images were collected for each subject, and the first image (T0) was taken immediately after the subject sat down or lay on their back with bare feet and legs extended forward and their feet off the ground. The second photo was taken five minutes later (T5), with the subjects in the same resting position.

**Figure 6:**
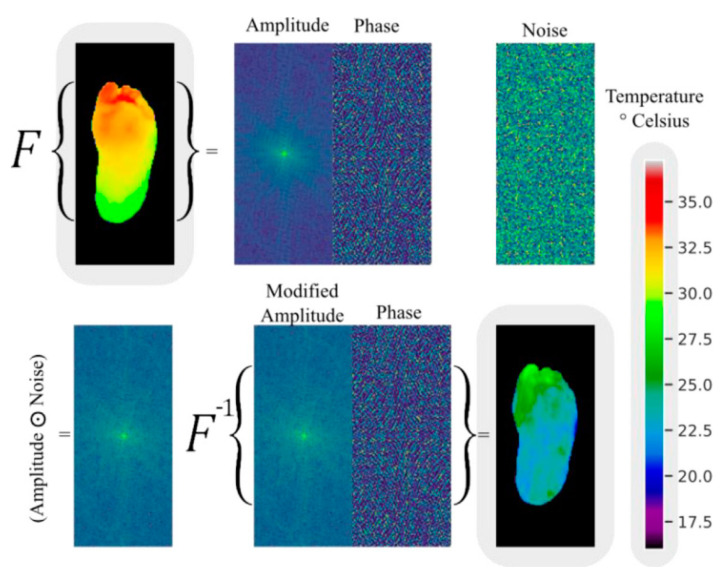
An example of data augmentation by Fourier transform [34].

Andres Anaya-Isaza and colleagues proposed a novel method for enhancing images based on variations in Fourier transform amplitude [34] (Figure 6). This method involves generating random images with uniform values ranging from 0 to 1, which are the same size as the amplitude matrix in the Fourier transform (referred to as noise images). These noise images are then subjected to a Hadamard product with the amplitude matrix, resulting in a modified amplitude matrix. Finally, an inverse Fourier transform is performed using this modified amplitude and the original phase to obtain the image in the spatial domain. In the evaluation of test data, the ResNet50v2 network achieved 100% accuracy, sensitivity, and specificity under the data augmentation condition of the combination of Fourier transform and image flipping. The DFTNet reached an accuracy of 97.06% with Fourier augmentation, and the Transformer had an accuracy of 88.24% without using Fourier transform and image flipping. From the distribution of metrics of the three models under different data augmentation conditions, it can be seen that flipping plus Fourier made the models exhibit higher stability and accuracy, and overfitting did not occur. 

#### 2.2.2. Transfer Learning

Transfer Learning (TL) is an approach that applies previously learned knowledge from a model trained on a specific task to another ML project. Fine-tuning pre-trained CNNs rather than full training has notable advantages, such as not requiring a large labelled dataset to extract and learn robust features for classification [35]. The functionality and significance of transfer learning in model training will be expounded upon in Chapter 3.

## 3. Processing Methods for Thermal Image of Diabetic Foot Ulcers

**Figure 7:**
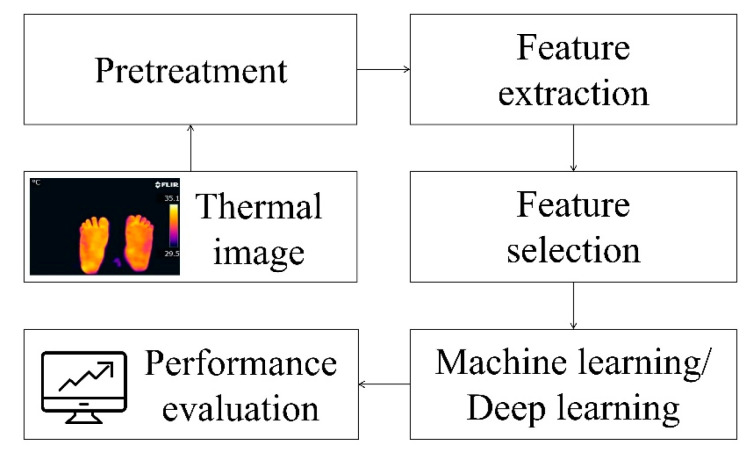
Processing steps for thermal imaging of diabetic foot ulcers [16,43].

Using infrared thermal imaging (IRT) to capture diagnostic thermal images to examine the temperature distribution of the feet and evaluate thermal changes is a fast, non-invasive, and non-contact method. Figure 7 summarizes the detection steps used in most existing studies.

### 3.1. Image Preprocessing

**Figure 8:**
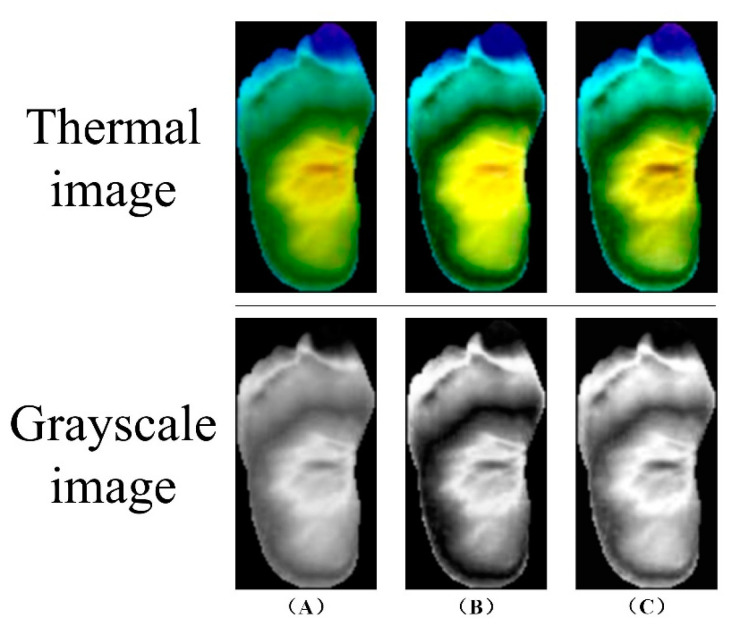
Image preprocessing: (**A**) Original image (**B**) Histogram equalization (**C**) Adaptive histogram equalization with limited contrast [7].

Image preprocessing is a crucial step in processing thermal images of DFU. It aims to improve image quality, enhance features, and provide better data for subsequent analysis. Common preprocessing techniques include histogram equalization, which enhances image contrast by redistributing pixel values, and adaptive histogram equalization, which performs contrast enhancement on local regions to avoid over-enhancement and noise amplification. Figure 8 demonstrates these preprocessing techniques.

### 3.2. Machine Learning-Based Methods

Image classification is an important task in disease diagnosis. Research on infrared thermal imaging in the field of DFU aims to classify images into binary or multiple categories. Various machine learning models, such as multilayer perceptrons (MLP), support vector machines (SVM) [36], logistic regression, and deep learning algorithms like AlexNet, GoogleNet, ResNet, MobileNet, and ANN [37] have been used for classification tasks. Traditional automatic classification techniques like artificial neural networks and support vector machines, which have common features such as parallel multiprocessing and data conversion using the kernel, provide high-accuracy results, especially in image classification and processing [38]. However, they require preprocessing and subsequent feature extraction and selection. Recently, attention has shifted to deep learning, achieving high-accuracy results through large-scale learning structures, enabling deep learning to capture deeper data features. Integrated deep learning models have been applied to binary classification tasks, such as infection versus non-infection and ischemia versus non-ischemia [39]. Furthermore, identification plays a crucial role in classification tasks. The identification process can extract useful features from images or data and match them with known categories to classify the input data [40]. Medical image segmentation can also serve as a multimodal information tool to assist in classifying ulcerated feet by distinguishing regions of interest (ROI) in the images, such as foot ulcers [41] and inflammation [42].

**Figure 9:**
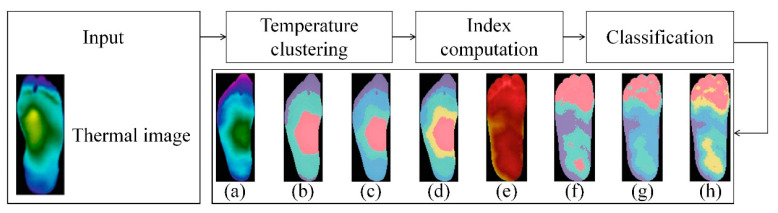
Demonstration of k-means algorithm and the results using different numbers of clusters [43]. Input thermal image and then acquire the results by means of temperature clustering, index computation and classification: (**a**) Original thermogram, non-diabetic. (**b**) From (**a**), 3-cluster result. (**c**) From (**a**), 4-cluster result. (**d**) From (**a**), 5-cluster result. (**e**) Original thermogram, diabetic. (**f**) From (**e**), 3-cluster result. (**g**) From (**e**), 4-cluster result. (**h**) From (**e**), 5-cluster result.

Filipe et al. [43] analyzed the diversity of thermal changes in the plantar region through foot thermograms of diabetes and healthy individuals in 2020, used K-means algorithm with different temperature values to divide the foot into five different clusters, and extracted features from the foot thermogram image [25] (Figure 9). Then, they carried out binary classification, and only for diabetes cases, multi class classification was carried out using SVM, weighted k-NN, logical regression, etc. Using the SVM quadratic model, the accuracy of binary classification is 0.980, and the accuracy of multi class classification is 0.966.

**Figure 10:**
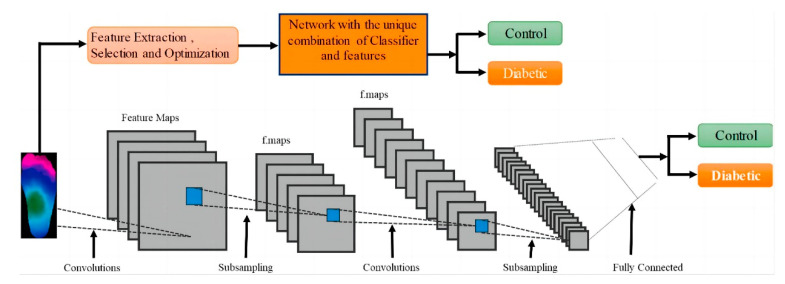
System diagram: Classification channels using one-dimensional (1D) and two-dimensional (2D) machine learning classifiers [26].

Amith Khandakar et al. explored various machine learning methods for classifying thermograms using feature engineering [26] (Figure 10). They used thermograms as input to extract, optimize, and rank important features. The best combination of top-ranked features was then used as input for classifiers, categorizing thermogram images into diabetic and control groups. Since the dataset is small, pre-trained models, originally trained on the ImageNet database were used. Through transfer learning, these models can utilize the feature representations learned from large-scale data, better adapt to the classification task of diabetic foot thermogram images, and effectively improve the generalization ability and detection performance. Furthermore, various image enhancement techniques were applied to improve two-dimensional thermographic images and enhance the performance of two-dimensional convolutional neural networks (2D CNNs). The performance of this approach was compared with traditional image classification models based on 2D CNNs, and the results indicated that classical machine learning classifiers performed very well. Specifically, MobileNetV2, a relatively shallow CNN model, achieved an F1 score of 95% in the classification of 2D thermographic images, while the AdaBoost classifier, using 10 features, achieved an F1 score of 97%. Additionally, the SVM classifier demonstrated an accuracy of 90.8%.

**Figure 11:**
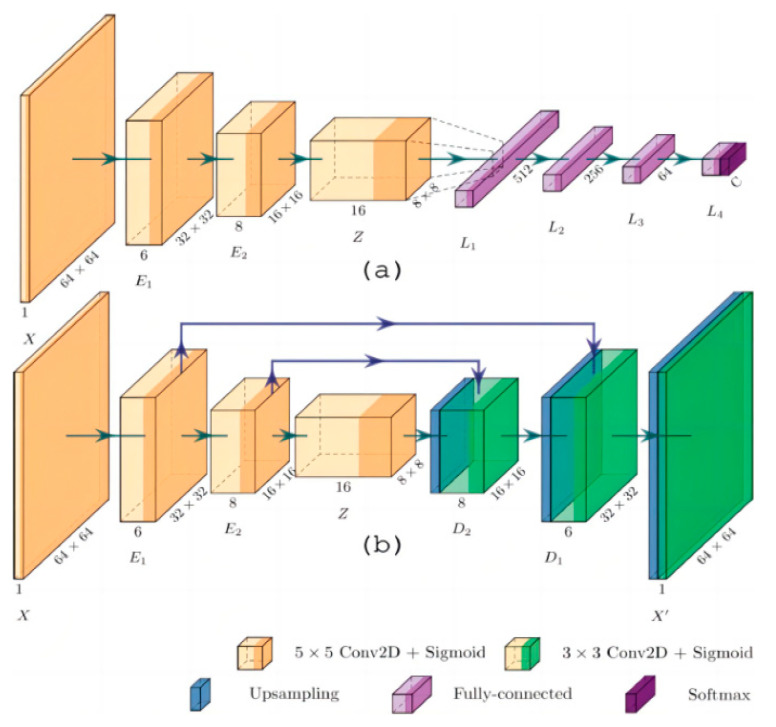
Network architecture: (**a**) Architecture for classification, with convolutional layers provided by pre-trained AE; (**b**) Proposed convolutional acoustic emission with optional skip connections [33].

Hernandez-Guedes A et al. proposed an innovative method to improve the performance of deep learning models when labeled data is scarce [33] (Figure 11). Through several experiments, they classified samples in a plantar thermogram dataset, enabling the early detection of diabetic foot ulcers. The proposed network architecture demonstrated superior results in classification metrics, making it effective for image classification on small datasets without relying heavily on commonly used classification metrics.

**Figure 12:**
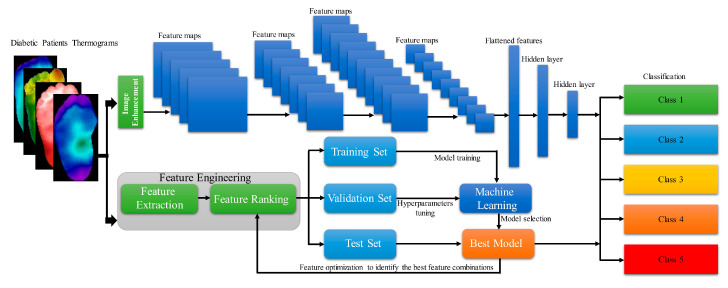
Demonstration of General Framework of Calculation Workflow [44].

Amith Khandakar et al. developed and proposed a general framework for multi-class (*n* = 5) classification of thermographic images, aimed at further enhancing classification performance [44] (Figure 12). They employed a variety of image enhancement techniques, including adaptive histogram equalization (AHE) and Gamma correction, along with classical machine learning algorithms, to process the thermographic images. The features extracted from these thermograms were then ranked and utilized as inputs for different two-dimensional convolutional neural network (2D CNN) models. The multilayer perceptron (MLP) classifier, along with features extracted from the thermographic images, demonstrated an accuracy of 90.1% in multi-class classification.

**Figure 13:**
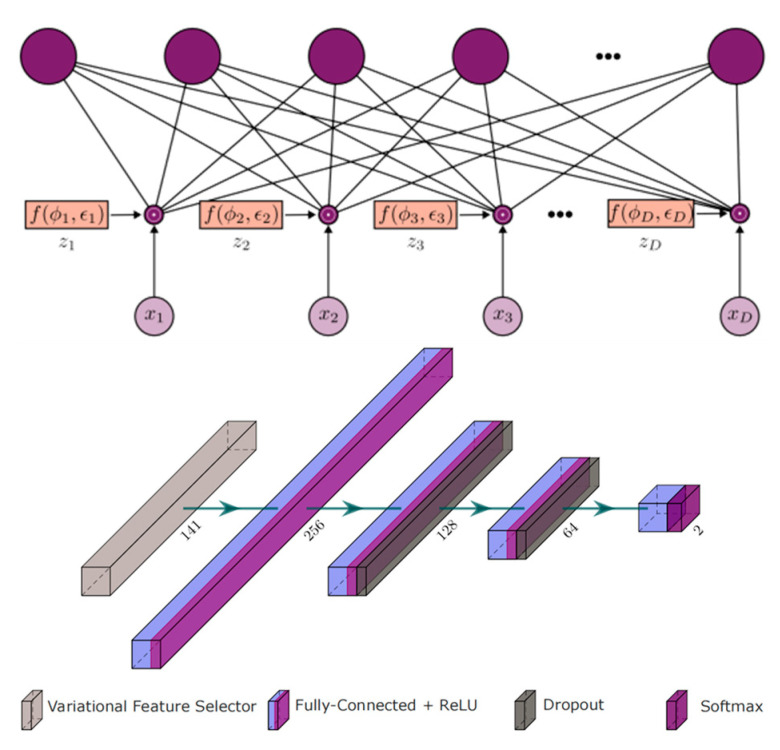
The Deep Learning Architecture Based on Dropout and Variational Dropout Method Feature Selection [45].

In 2023, Abian Hernandez-Guedes et al. merged the INAOE dataset with local thermograms from healthy volunteers and used Synthetic Minority Over-sampling Technique (SMOTE) to generate synthetic data, thus expanding and balancing the database [45] (Figure 13). They employed classical methods such as LASSO regression and Random Forest, as well as advanced deep learning-based techniques (specifically, dropout and variational dropout) to extract state-of-the-art features. The most relevant features were then identified and ranked. The dropout’s variational technique can be interpreted as an embedded feature selection algorithm. The variational parameter *φd* determines the likelihood of the input feature *xd* being discarded, and it is utilized for feature ranking. These extracted features were used to classify subjects at risk of ulcers, with a support vector machine (SVM) classifier with fixed hyperparameters serving as a reference to evaluate the robustness of the selected features. Among the implemented extraction methods, the variational dropout technique performed best, achieving an F1 score of 90% using the SVM classifier. This represented an approximately 15% improvement in classification performance compared to previously regarded state-of-the-art features.

## 4. Evaluation Metrics

To effectively evaluate the performance of diabetic foot ulcer (DFU) diagnostic methods using thermal imaging, a variety of well-established metrics are employed to assess the accuracy, reliability, and generalizability of these models. Each metric offers insights into different aspects of model performance, helping researchers and clinicians understand both the strengths and limitations of the diagnostic approach.

Sensitivity (True Positive Rate, *TPR*): Sensitivity measures the proportion of actual DFU cases correctly identified by the model, focusing on the model’s ability to detect true positives. A high sensitivity rate is crucial in early screening, as it is more important to correctly identify a diabetic foot ulcer than it is to incorrectly identify a non-ulcerated area [46]. In clinical settings, achieving high sensitivity is essential to minimize the risk of undetected DFU cases, which could progress and result in severe complications if left untreated.

Specificity (False Positive Rate, *FPR*): Specificity evaluates the model’s accuracy in identifying healthy, non-ulcerated feet by measuring the proportion of true negatives. A high specificity rate indicates that the model effectively distinguishes between DFU and non-DFU cases, reducing the likelihood of false positives, which can lead to unnecessary patient anxiety and additional testing. Balancing sensitivity and specificity is critical in achieving a reliable diagnostic system, as an overly sensitive model may yield excessive false positives, while a model with high specificity may miss true DFU cases. In personalized treatment, high specificity is of particular importance as it aligns with the aim of precisely tailoring medical approaches to individual patient conditions and helps avoid subjecting patients to inappropriate or unnecessary treatments by minimizing false positives.

F1 Score: The F1 score is a harmonic mean of sensitivity and precision, offering a balanced measure of the model’s accuracy, especially when dealing with imbalanced datasets. This score is particularly valuable in DFU detection, where infected and ischemic cases may be unequally represented. By taking both false positives and false negatives into account, the F1 score provides a more comprehensive evaluation of the model’s diagnostic performance.

Precision: Precision, or positive predictive value, assesses the proportion of true positive DFU detections among all positive predictions made by the model. High precision is important for reducing false positives, ensuring that when the model flags a foot as potentially ulcerated, it is likely to be correct. This metric is essential in resource-limited settings where unnecessary interventions can strain healthcare resources.

Area Under the Curve (AUC): AUC represents the area under the receiver operating characteristic (ROC) curve, which plots the model’s sensitivity against 1-specificity across various threshold levels. A high AUC score indicates a good balance between sensitivity and specificity, reflecting the model’s overall ability to discriminate between DFU and non-DFU cases. AUC is widely used in diagnostic studies as it provides a single metric summarizing model performance across thresholds, aiding in the selection of the optimal decision threshold for clinical use.

Overlap Error: Overlap error measures the degree of misclassification by assessing the overlap between predicted and actual thermal regions associated with DFUs. This metric is particularly relevant in image-based diagnostics, where models must accurately localize affected areas to guide clinical assessment. Lower overlap error values indicate a more precise identification of DFU regions, supporting accurate diagnosis and tailored intervention.

Accuracy: Accuracy is a general metric representing the proportion of correctly classified cases (both DFU and non-DFU) among all cases. While accuracy provides a straightforward measure of performance, it may not fully capture the effectiveness of the model in scenarios with imbalanced datasets, as it can be disproportionately influenced by the majority class.

These metrics are calculated using fundamental components of classification—true positives (*TP*), true negatives (*TN*), false positives (*FP*), and false negatives (*FN*) [47]:

(1) $$Accuracy=\frac{TP+TN}{TP+TN+FP+FN}$$

(2) $$TPR=\frac{TP}{TP+FN}$$

(3) $$FPR=\frac{FP}{TP+FP}$$

(4) $$F1\text{\_}Score=\frac{2TP}{2TP+FP+FN}$$

(5) $$Specificity=\frac{TN}{TN+FP}$$

where, true positives represent correctly identified DFU cases, while true negatives are non-DFU cases accurately identified as healthy. False positives occur when non-DFU cases are incorrectly classified as DFU, and false negatives happen when DFU cases are missed by the model. By combining these elements, each metric offers unique insights into the diagnostic model’s strengths and areas for improvement, supporting the development of robust and clinically applicable DFU detection systems.

Table 1 provides a comparative summary of the performance achieved by various machine learning models in the classification and detection of diabetic foot ulcers (DFUs) using thermal imaging. The models evaluated span a range of machine learning approaches, including traditional classifiers such as Support Vector Machines (SVM), advanced convolutional neural networks (CNNs), and customized architectures like DFTNet. These models have been developed and tested within the past three years, reflecting the latest advancements in the field of DFU diagnosis.

The results indicate that different models excel in various aspects of classification performance. Custom CNN architectures achieved the highest overall accuracy, demonstrating their ability to learn and identify complex thermal patterns associated with DFUs effectively. CNNs, with their deep learning capability, excel in processing large volumes of image data, extracting detailed spatial features, and distinguishing between fine temperature differences, making them particularly suited for DFU detection. Custom CNNs also achieved the highest F1 score, indicating a strong balance between sensitivity and precision, which is critical for reducing both false positives and false negatives in clinical diagnoses.

Support Vector Machines (SVMs), widely known for their robustness in classification tasks, achieved the highest sensitivity, indicating their ability to identify a high proportion of true DFU cases. This is particularly important in clinical settings, as high sensitivity ensures that most DFU cases are detected, reducing the likelihood of missed diagnoses that could lead to severe complications. SVMs are often favored for their effectiveness with smaller datasets and their ability to handle high-dimensional data, making them a valuable choice for thermal imaging applications where labeled data may be limited.

DFTNet, another advanced architecture tailored specifically for DFU detection, exhibited the best specificity, highlighting its strength in accurately identifying non-DFU cases and minimizing false positives. High specificity is essential for avoiding unnecessary interventions and ensuring that only patients at genuine risk of DFUs are flagged for further examination. DFTNet’s performance suggests that customized networks trained on domain-specific features—such as foot thermography patterns associated with DFUs—can outperform general-purpose models in achieving specialized diagnostic goals.

The diverse strengths of each model highlight the importance of selecting the appropriate method based on specific clinical needs and diagnostic objectives. For applications where sensitivity is paramount, such as screening programs aimed at early detection, models like SVM may be preferred. In contrast, settings that prioritize overall diagnostic accuracy and balanced performance across metrics might benefit from using Custom CNNs. Meanwhile, for cases where false positives need to be minimized to prevent unnecessary treatment, models like DFTNet with high specificity would be ideal. 

As DFU detection continues to evolve, this comparative analysis of model performance underscores the potential for developing hybrid approaches that combine the strengths of multiple algorithms, optimizing both sensitivity and specificity to create robust, clinically adaptable solutions for DFU diagnosis and management.

## 5. Future Challenges

Although infrared thermal imaging (IRT) is a rapid, non-invasive, and contact-free method for detecting diabetic foot ulcers (DFUs), several challenges remain in its application for early warning and prevention. While thermal imaging provides valuable insights into temperature variations that may indicate neuropathy, ischemia, or inflammation, achieving reliable and clinically applicable results requires overcoming specific technical and methodological hurdles.

Data Quality: The quality of DFU thermal images can vary significantly, as these images are often collected from diverse sources and labeled by clinicians with varying levels of expertise. This variability in data quality and labeling consistency poses a major challenge for machine learning algorithms, which rely heavily on accurate, high-quality data to make precise predictions. Differences in image resolution, lighting, and equipment settings can introduce anomalies or noise in the data, reducing model reliability. Furthermore, clinicians’ subjective interpretations may lead to inconsistent labeling, especially when distinguishing subtle temperature differences indicative of early-stage DFUs. Developing robust detection algorithms that can accommodate these inconsistencies is crucial to accurately predict disease onset and progression. Advanced preprocessing techniques and quality assessment protocols are essential for ensuring the data used in training and testing remains reliable, representative, and standardized across multiple sources.

Data Imbalance: Publicly available DFU datasets are often highly imbalanced, with a significant number of infected cases but relatively few ischemic cases. This imbalance in class distribution can hinder the performance of machine learning models, as they may become biased towards the majority class (i.e., infected cases) and perform poorly on minority classes (e.g., ischemic cases). This issue is particularly problematic for early DFU detection, as ischemic conditions, while less frequent, require timely intervention to prevent serious complications. To address data imbalance, Generative Adversarial Networks (GANs) and other data augmentation techniques can be applied to generate synthetic images that resemble real ischemic cases. By artificially increasing the representation of ischemic cases, GANs help create more balanced datasets, enabling models to learn features associated with each condition more effectively. This approach not only enhances model performance across classes but also improves generalizability, ensuring that detection systems can reliably identify a broader spectrum of DFU presentations.

Handling Multiple Labels: A critical challenge in DFU detection is accurately handling cases with multiple concurrent conditions, such as ischemia and infection. Each condition affects foot temperature in distinct ways: ischemic cases are generally associated with cooler regions due to reduced blood flow, while infected neuropathic cases present as warmer areas due to inflammation. When these conditions occur simultaneously, as they often do in advanced DFUs, identifying and distinguishing them becomes highly challenging. Current machine learning models frequently struggle to interpret mixed signals, as thermal variations associated with one condition may mask or alter those of the other. Future detection techniques should consider multi-label classification approaches that can handle these overlapping conditions, allowing models to identify and categorize different pathological states within a single thermogram. Such multi-label frameworks would enhance diagnostic precision and provide clinicians with more comprehensive insights into patients’ foot health, supporting more targeted and effective treatment plans.

Environmental impact: Environmental temperature fluctuations can significantly affect the accuracy of thermal imaging results. Sudden changes in temperature, whether an increase or decrease, may cause false positives or negatives, as the device may struggle to differentiate between normal physiological variations induced by environmental stress and true pathological conditions. In addition, lighting conditions present another challenge. Intense sunlight or strong artificial light sources can introduce extraneous heat or reflections, which interfere with the thermal imaging sensor’s ability to capture accurate thermal patterns. This interference is especially problematic when detecting small or superficial lesions, as the heat or light-induced artifacts can obscure subtle thermal variations associated with these pathologies.

In summary, while infrared thermal imaging holds immense potential for early DFU detection, addressing issues related to data quality, class imbalance, multi-condition labeling, and environmental impact is essential for its broader adoption in clinical settings. Overcoming these challenges through advanced image preprocessing, data augmentation, and sophisticated machine learning techniques can significantly improve the reliability and utility of IRT, ultimately making it a standard tool for DFU prevention and management in diabetic care.

## 6. Conclusions and Perspectives

Diabetic foot ulcer is a preventable chronic disease, yet if not treated timely, it can lead to repeated hospitalizations, limb or toe amputations, and even death in severe cases. Early symptoms of DFU are not apparent, making early diagnosis challenging. However, with continuous advancements in image processing technology, thermal imaging, with the aid of intelligent image interpretation, is becoming an important tool for early detection of DFU.

This review first introduces the pathogenesis and urgency of early diagnosis of DFU. It further discusses thermal imaging technology as a non-invasive method with potential usage for early identification, classification, and diagnosis of DFU. The paper highlights the mainstream open datasets, widely used data augmentation, preprocessing, and modeling methods, and discusses optimizations to improve model performance. Detailed image processing methods for DFU thermal images are elaborated, emphasizing the importance of preprocessing and machine learning-based methods in this field. Finally, the review introduces the evaluation metrics for DFU diagnostic methods, discussing the strengths and limitations of various existing methods and proposing the challenges of using infrared thermal imaging for DFU early warning.

Looking ahead, as image processing technology continues to advance, thermal imaging will be more widely applied in the identification of DFU. With ongoing research efforts, this methodology is expected to achieve breakthrough progress to serve as a standard and important toolset in early warning and treatment of DFU.

## Figures and Tables

**Table 1:** The performance of various existing work using thermal images of diabetes foot ulcer for classification.

Method	Accuracy	TPR	FPR	F1 Score	Precision	Ref.
DFTNet	0.9453	0.9534	0.9375	0.9457	0.9401	[48]
DenseNet201	0.9401	0.9401	0.9078	0.9401	0.9401	[26]
Custom CNN	0.9827	-	-	0.9621	0.9626	[32]
SVM + SURF	0.9781	-	-	-	-	[49]
SVM Quadratic	0.912	-	-	0.825	0.826	[50]
CNN + ML	0.91	-	-	0.91	0.91	[44]
K-means	0.742	0.7300	0.778	0.805	0.899	[43]
SVM	0.9561	0.9650	0.9241	-	-	[51]
FC + fancy PCA	0.985	-	-	0.9896	1.00	[35]
SVM + fancy PCA	0.985	-	-	0.9898	0.98	[35]
LR + fancy PCA	1.00	-	-	1.00	1.00	[35]

## Data Availability

Data are contained within the article.
